# Medicina Forensica

**Published:** 1828-01-01

**Authors:** 


### Medicina Forensica.

### 1. THE COLLEGE OF PHYSICIANS.

" Periturae parcite Chartae."

If the College or its partizans think that we have been too severe in our comments, or too extravagant in our demands for reformation, we beg they will calmly peruse the following document, drawn up and presented to the College 30 years ago, by 14 as eminent physicians as that Institution can boast of. In this remonstrance, petition, or whatever else it may be called, will be seen most of the leading principles for which we contend; and happy would it have been for the College, if it had hearkened to the reasonable request of the Licentiates thirty years ago, instead of having recourse to measures which we shall be compelled reluctantly to portray in our next number, should no appearances of better feelings be developed in the interim.

" *To the President and Fellows of the College of Physicians of London:*

" Gentlemen,

" We, whose names are subscribed, licentiates of the college of physicians of London, conceiving ourselves *unjustly* deprived of those professional honours and privileges to which we are entitled, and persuaded that every physician of irreproachable character and of competent learning has a legal right to be admitted a fellow of the college, submit to your dispassionate consideration the following grounds of this conviction.

" In the beginning of the sixteenth century, when learning had made but little progress in this country, and medicine was chiefly practised by ignorant empirics, the physicians of London were incorporated into a *college or community*, by a charter from Henry VIII. The object of the grant, as declared by the charter, was to provide for the safety of the subjects of the realm, by restraining the audacity of those wicked men, who practise medicine more from avaricious motives than from any good intention, to the great injury of the illiterate and credulous multitude. With this view, partly in imitation of well regulated states in Italy and many other countries, and partly in compliance with the prayer of John Chambre, Thomas Linacre, Fernando de Victoria, the king's physicians, of Nicholas Halsewell, John Francis, and Robert Yaxley, physicians, and of Cardinal Wolsey, a perpetual college of discreet and learned men was established, consisting of the above-named six physicians, and *all other men of the same faculty*, residing in London. The members of this body were authorised to exercise their profession in London and its neighbourhood, and, at the same time, were strictly required to prevent every other person from practising medicine, either in that city or within seven miles round it, unless he had been admitted to do this by the college; and in order that this injunction might be carried fully into effect, they were invested with powers to punish the disobedient.

" It is evident then, that neither the graduates of Oxford and Cambridge, nor those of any other university, were to derive any partial advantages from the charter; but on the contrary, that *all* discreet and learned physicians, residing in London at the time it was granted, were indiscriminately admissible into the college, and that the perpetuity of the body was to be preserved by the reception of *every* physician of the same description, who should ever practise medicine in London, or its vicinity, and should claim to be a member. Can it be supposed, that Henry VIII. while he declared that the good of his subjects at large was the object of his grant, *in reality meant to*

*benefit only a few, to the prejudice of many; or, that he thought physicians of learning and good morals were to be found, exclusively, among the graduates of Oxford and Cambridge, though the only physicians he himself employed had taken their degrees abroad?* Can it be for a moment imagined, that the principal physicians who obtained the charter, *all of them foreign graduates*, could intend, that none under the like circumstances should *ever after* have a right to claim admission into the college; or, that if either their sovereign or themselves had entertained such a design, this would not have been expressly declared?

"But had any obscurity existed in the language of the charter, with respect to the description of persons to be admitted into the college, or had experience discovered, that the qualifications required by it were inadequate to the attainment of the object of the grant, ample opportunities were afterwards afforded of explaining what was doubtful, of amending what was wrong, and of supplying whatever had been found wanting. Four years after the date of the charter, while the sovereign who bestowed, and the six physicians who obtained it, were still living, an act of parliament was passed, in consequence of a petition from the same physicians, confirming the old, and conveying new privileges to the college. *No change, however, was made by it in the requisites for admission.* Various other acts of parliament, of a still later date, relating to the college, are to be found in our statute books, but all of them are silent respecting the admission of members. Nor does there occur any new article upon this head, in the charters granted to the college by James I. and Charles II. *We have therefore the fullest evidence, that the only qualifications for admission into that body required by the charter of Henry* VIII. *were learning and good character, and that the various parliaments which confirmed that charter, and the different sovereigns who granted others, all concurred in regarding those qualifications as sufficient.* The college, indeed, for many years after their incorporation, did not demand any other. Not to accumulate proofs of this point, we shall only mention, first, that it appears from Seymour, in his Survey of London, that in 1575, fifty-seven years after the grant of the charter, the college consisted chiefly of persons who had graduated abroad; secondly, that one of the ancient statutes of the college expressly relates to the admission of foreign graduates: the statute is, 'Quoniam multi huc con-'fluunt, &c. idcirco statuimus, ut, qui-'cunque vel in *collegii societatem*, vel in '*candidatorum* ordinem, vel in permisso-'rum numerum admittetur, si doctoratus 'gradum *apud exteros* susceperit, is, ad-'missionis tempore, duplo plus solvat, '&c. quam illi solvere solent, qui *in nos-'tris academiis* doctores creantur:' and lastly, that the following statute respecting the form of examining *fellows* and *candidates* was made by the college in 1647, and was not repealed till 1736: 'Si doctoris gradum in aliquâ *nostrarum* 'academiarum susceperit, honoris causa 'sedeat decenter examinandus, ne quid 'indignum pati a nostrâ examinationum 'formâ mater academia videatur;' a regulation which clearly implies that foreign graduates might also be examined as candidates for the fellowship.

"Should it be objected to what has here been advanced, that those physicians who had taken degrees abroad, were obliged, before they were received into the college, to be incorporated to a degree at Oxford or Cambridge, we would in reply maintain, that this practice was not adopted, till some time after the foundation of the college, and *that the bye-law which gave rise to it was illegal.* But how was incorporation in medicine at the English universities formerly obtained? On this point, Dr. Winterton furnishes ample information. In a letter to Dr. Foxe, president of the college of physicians of London, dated so lately as in 1635, he writes, 'I have observed and grieved to see, sometimes a minister, sometimes a serving man, sometimes an apothecary, admitted to a licence to practise in physic, or to be *incorporated to a degree*, without giving any public testimony of his learning and skill in the profession;' and in another part of the same letter he says, that *incorporation* was 'in an instant obtained by a little sum of money.' Such an incorporation could surely furnish no proof of the learning or character of a candidate for admission into the college; and the only motive for requiring it, seems to have been a desire in the members to increase the emoluments of the universities, where many of themselves had been educated.

"The act of parliament passed in the fourteenth year of Henry VIII. ratifying the charter of the college, indeed grants *one* privilege to those physicians, who had taken their degrees at Oxford or Cambridge, without any grace; namely, that of practising medicine in any part of the kingdom, except London, without being subject to an examination before the college. But this very grant confirms our claim, for since a particular privilege is specified in the act, *it is clear the legislature intended that those graduates should derive no other from it.*

"It may be said, however, that although neither the crown, nor the legislature, has conferred any privilege upon the graduates of Oxford and Cambridge, with respect to admission into the college, still the members of the college themselves have a right to make a distinction in this respect, in favour of those graduates, by virtue of the power to frame bye-laws which their charter confers upon them. To this we answer, that it is an established maxim in law, that the power in corporations to make bye-laws extends only to matters of regulation. *They can create no new rights, nor take away any of those which are conveyed by the charter of incorporation. They can change neither the object of the institution, nor the nature of the succession.* Every candidate included in the meaning of a corporate grant, and possessing the qualifications required by it, of whatever nature they may be, has an undoubted right to an examination of his claim, which, if it prove satisfactory, must be admitted.

"The arguments we have urged we shall now support by the authority of men, long conversant in the interpretation of ancient records, of the most profound knowledge of the laws of our country, and above all suspicion of prejudice or partiality; of men, not giving their opinions casually or lightly, but solemnly delivering them in their high characters as judges, upon a subject they had maturely considered.

"In the case of the licentiates against the censors of the college (Burrow's Reports, Vol. IV.) Lord Mansfield delivered the following opinions:

"'The main end of the incorporation was to keep up the succession, and it was to be kept up by the admission of fellows after examination. *The power of examining,* and admitting after examination, was not an arbitrary power, but *a power coupled with a trust.* They are *bound* to admit *every person* whom on *examination* they think *fit* to be admitted, within the description of the charter, and the act of parliament which confirms it. The person who comes within that description has a *right* to be admitted into the *fellowship.* He has a claim to several exemptions, privileges, and advantages attendant upon admission into the fellowship; and not only the candidate himself, if found fit, has a personal right, but the *public* has also *a right to his service,* and that not only as a physician, but as *a censor,* as *an elect,* as *an officer* in the offices, to which on admission he will become eligible.'

"'The charter and statute have left every thing at large to the college, no way confined or restrained but by the *fitness* of the objects.'

"'I think that every person of proper education and requisite learning and skill, and possessed of all other due qualifications, is entitled to have a licence, and I think he ought, if he desires it, to be *admitted into the college.*'

"'It has been said, that there are many among the licentiates who would do honour to the college by their skill and learning, as well as other valuable and amiable qualities, and that the college themselves, as well as every body else, are sensible that this is in fact true and undeniable. If this be so, how can any *bye-laws* which exclude the possibility of admitting such persons, stand with the *trust reposed in them of admitting all that are fit.*'

"In the trial of Dr. Letch, Mr. Justice Aston, after quoting part of the act of parliament of the fourteenth of Henry VIII. which confirms the charter said; 'This shews that the makers of the act looked upon those of the faculty residing in London to be members of the college.' He added, that if the college should refuse to examine a candidate, a mandamus would lie. It was also his opinion, that in grants of this kind, the construction ought to be made in a liberal manner.

"Mr. Justice Yates in the course of the trial of the licentiates against the censors, expressed himself thus: 'A good deal has been said about *long usage*. But usage only applies when the *construction is doubtful*. Here the construction is *not doubtful*. If it were, then indeed usage for two hundred years might have weight.'

" It appears then fully established by the declared object of the charter, the description of persons first incorporated, the usage of the college for a considerable time after their incorporation, and the opinions delivered by Lord Mansfield and Mr. Justice Aston, with the concurrence of the other judges who sat with them on the bench, that the college are *bound* to examine every respectable physician who desires to be received into their body, and, if found possessed of requisite learning and skill, *that they are under both a legal and moral obligation to admit him a member of the community*. But if the right of admission belongs to *every* respectable physician of competent knowledge, *we* certainly cannot, consistently either with our honour or our interest, tamely submit to be excluded. Our opportunities of acquiring the knowledge of our profession have been equal to those of the best educated physicians in this kingdom. In the choice of universities we have been determined by the celebrity of the teachers; and wherever superior means of improvement were afforded, either at home or abroad, many of us have availed ourselves of them. *If our talents therefore be not inferior, the proportion of persons of considerable medical learning and skill among us, must be equal to that which exists in any other body of physicians. We appeal then to the candour and justice of the college, whether, if we even possessed no* legal *title to what we claim, when our number, education, acquirements, and conduct are considered, it be not reasonable that we should participate in the distinctions and advantages of an institution, established by the legislature for* THE PUBLIC GOOD, AND THE ADVANCEMENT OF OUR PROFESSION.

" The college cannot feel more strongly than we do the necessity of being cautious with respect to the members they admit, or be more anxious that unimpeached morals and competent learning should be the tests of eligibility. Nor should we object to any other regulations that might secure the utility and dignity of the institution, provided they were impartially extended to every candidate before, and to every member after admission. *But we cannot rest satisfied with the present mode of introducing licentiates through favour; because it implies, on our part, a defect of right, and inferiority of qualification; because it is precarious, and subjects many of our body to be contemptuously passed by, and consequently to be depreciated in their characters, and wounded in their feelings; because it* ENCOURAGES SERVILITY TOWARDS THE COLLEGE, *and* COMPETITION *and* ANIMOSITY *among ourselves; and finally, because it exposes us, even after admission into the community, to be considered, by some at least, as intruders, who owe to the bounty of patronage, what ought to be the reward of merit.*

" At this period, when ignorant and unprincipled empirics so greatly infest the capital, and when, perhaps, the severe measures once exercised, with respect to such men, are no longer practicable, does it not become the especial duty of the college to draw the strongest possible line of distinction between the upright and skilful physician, whose interest the legislature designed to promote, and the daring impostor it meant to restrain and to punish?

" This application arises from no hasty project, or restless spirit of innovation. It is meant to advance a claim, which, we are well warranted to believe, is founded both in law and in equity, a claim repeatedly urged by our predecessors, who, we are convinced, only failed in their attempts to establish it from the injudicious measures they pursued.*

" We conclude this address, by acquainting you, that several of our number, against whom no objection can be made in respect of morals or conduct, are desirous to submit to your decision, their claim, as men of learning and skill in their profession, to be members of the college; and by requesting to know, whether, upon application being made by any such person for an examination, previously to his being a candidate for a fellowship, you will admit him to the same trials, which the graduates from Oxford

* We beg leave to refer our readers to " An EXPOSITION of the State of the Medical Profession, &c." published by Longman and Co. 1826, pp. 373.

and Cambridge in like cases undergo; and whether, if his learning, and skill, and character, be approved, you will receive him into the college upon the same terms, and in the same manner, as if he had graduated at either of those universities.

| | |
|---|---|
| *John Cooke.* | *Samuel Ferris.* |
| *W. C. Wells.* | *Sayer Walker.* |
| *Christr. Stanger.* | *Wm. Woodville.* |
| *Edward Fryer.* | *John Relph.* |
| *Alex. Crichton.* | *John Hemming.* |
| *James Sims.* | *Lawrence Nihell.* |
| *John Aikin.* | *Wm. Hamilton."* |

26th June, 1795.

To the above address, the College gave no answer; and the Licentiates, finding that nothing could be obtained from the equity of the Corporation, proceeded to the recovery of their privileges by law. Of the proceedings which ensued, and over which, it would be well for the College that the veil of oblivion were for ever drawn, it will be our painful duty to speak in our next number.

### II.

### MEDICAL EDUCATION.

The Physical Society of Guy's Hospital opened on Saturday night, the 6th of October, with a most animated discussion of a paper read by Dr. Hodgkin, on Medical Education. The theatre was crowded to excess, and many of the most talented members of the profession were present as visiters.

Dr. H.'s paper was almost entirely dedicated to the subject of the student's education subsequent to the completion of the apprenticeship, as he did not think that the preceding portion of the student's time came properly under his (Dr. H.'s) cognizance. The Society, however, thought otherwise, and the whole evening was occupied with arguments for and against apprenticeships. Dr. Addison opened the campaign, and made a most brilliant and powerful speech. He placed in the most striking point of view the absurdity of that law, which condemned the young man to sacrifice *five years* of his life—say from 15 to 20—to the drudgery of pounding and compounding drugs, whose chemical properties and medicinal agencies he was entirely ignorant of, while the short space of 18 months was considered sufficient for acquiring the whole range of the medical sciences afterwards! He did not wish to throw any blame on the Worshipful Company of Apothecaries. They had, doubtless, done all they could to improve the education, and, consequently, the utility and respectability of the general practitioner—and infinite good had unquestionably been done by the late Act of Parliament. But still the law of apprenticeship was bad—very bad—and most of the laws relating to the profession generally, were calculated to repress, rather than encourage medical science. He traced the bad laws of the Apothecaries' Company to a higher source than that of the Company itself—to the Royal College of Physicians—whose monopolizing statutes and illiberal restrictions were at once a nuisance and disgrace to the age we live in, and the country we inhabit. He said it was most deplorable that the ear of a monarch renowned for love of science, and the encouragement of literature, should be poisoned by men who ought to use their influence for the general good of the medical profession. Dr. A. indeed, drew a picture here, and made observations, which, though but too true, might yet endanger a prosecution, if we portrayed them on our pages. "The greater the truth, the greater the libel."

The same view of the subject of apprenticeships was taken by Dr. Birkbeck, who ably exposed the loss of time thus incurred. He maintained that apprenticeships of all kinds were bad—even in the most mechanical trades. He forcibly argued that, when the youth is taken from school, say at the age of 16 years, he should at once commence the acquisition of general knowledge, and the rudiments of his own profession. Natural philosophy—natural history—chemistry—materia medica—even anatomy, should be learnt during those years that are spent behind a counter.

Some others followed on the same side, among whom was Dr. Whiting. Mr. Hardy, Dr. Rees, Mr. Key, Mr. Johnson, and one or two others, made feeble attempts to support the system of medical apprenticeship; but their arguments were contradictory, puerile—and some of them laughable. Thus, Dr. Rees submitted that apprenticeship was necessary and useful, as affording the youth an opportunity of imbibing moral, religious, and domesticated habits, from the example of his master and mistress. Mr. Hardy instantly rejected this argument as perfectly worthless, inasmuch as it was the common practice of the apprentice to learn games of romps in the kitchen with the servants, rather than morality and domestic habits in the parlour. (Laughter.) All on this side of the question seemed to agree, that nothing else could be done with a youth of 15 years, than bind him apprentice for 5 years, to keep him out of harm's way! Mr. Hardy, indeed, while he half condemned and half approved the principle of apprenticeship, strongly urged the rising generation, when they became masters in their turn, to endeavour to inculcate habits of acquiring general knowledge, but especially mathematics, in the young men while under their charge. This gave origin to a rather caustic remark from Dr. Rees—namely, that he had never known a physician, celebrated for his knowledge of Greek, Latin, and mathematics, who was worth a farthing at the bed-side of sickness. The sticklers for Oxford and Cambridge, if any were present, did not choose to dispute this point. Dr. Rees excited a laugh when he alluded to the writings of the mathematical physiologists and physicians, some of whom proved to a demonstration, that the force of the heart was only equal to a few ounces, while others proved, by the same "exact science," that the force of the central organ of the circulation was equal to many thousand pounds!

There were some members who proposed a modification of the apprenticeship, which, they averred, had been already acted on with much advantage. This was, the permission to attend lectures the two first years of the apprenticeship, which rendered the pupil a very useful assistant to his master during the other three years. The general voice of this enlightened assembly was clearly against the present system—some wishing it abolished in toto, while almost all recommended an abridgment of the period of five years.

For our own parts, we have always been of opinion, and this opinion is grounded on actual experience, (having ourselves gone through the drudgery of an apprenticeship) that the actual state of things is bad in this respect. There are some masters, and there are some situations, in which the whole period of five or six years may be advantageously passed under indenture; but these are little more than exceptions to general rules. We conscientiously believe, that two-thirds, at least, of the period of servitude (for it is nothing else) are lost—or worse than lost. When the business of the apothecary was a mere matter of pounding, compounding, boiling, extracting, and distilling, as it now is with the chemist, there was some reason for indenting a youth for seven years to become *au fait* in all these various and complicated manipulations. But now, when the general practitioner is, to all intents and purposes, the general physician and surgeon—when his pursuits are intellectual, and not mechanical, it is absurd to cling to the old system of apprenticeship. True it is, that, under the existing order of things, there will be much inconvenience in dispensing with the services of the apprentice—for who is to make up the medicines, when the master comes home, tired with his daily rounds? We hope the day is not far distant, when the general practitioner will shake off entirely all connexion with the dispensing of medicines, and with the mere trading part of the profession. We hope and trust that we will yet live to see the day, when every general practitioner's medical education will be as complete—his studies as extended, and his examinations as rigid, as those of the physician and surgeon so called. This approximation is rapidly approaching—and, as it draws to a consummation, his medical attendance on the sick must and will be rewarded by fees, and not by the price of draughts and pills prescribed. He will then require no apprentice.

In the course of the discussion this evening, a very ludicrous *collision* took place among the three grades of the profession, which, at one time, threatened a WAR—of words at least! The brother of Dr. Hodgkin, (who, we believe, is of the legal profession) evidently with the best intentions, read some extracts from certain judicial records, shewing that the original *apothecary* united the very lucrative business of *grocer*, with the "art and mystery" of his own peculiar calling. This was like a spark of fire applied to a barrel of gun-powder! The enquiry was immediately instituted as to the origin of the *surgeon* ; when it was clearly proved that *he* did not, in days of yore, disdain to flourish the razor, and thereby drop the penny into the till, for smoothing the chin of the—" unwashed artisan," on a Saturday evening! *(Roars of laughter.)* The *physician's* turn came next. The younger Mr. Hardy shewed that " their high mightinesses" the cotemporary physicians of the barber-surgeons and grocer-apothecaries, united with the "art and mystery" of their avocations the sublime science of ASTROLOGY—and that many of them derived a larger revenue from the interpretation of what was passing among the *heavenly bodies*, than what was going on in the *bodies of their patients*. *(Continued laughter.)* There was now a kind of instinctive impulse to put an end to this investigation. The physicians, the *pure* surgeons, the general practitioners, and the " Worshipfuls," all jumped up, as it were, *per saltum*, to apologise to, and compliment each other on the *millenium* which now obtained in medical science! All retrospection of the past, and investigations into the origins of the different ranks in the profession were dropped by mutual consent—as subjects by no means calculated to promote the most pleasant feelings, or conduce to the harmony of the society.

There was an observation which fell from Mr. Johnson, one of the examiners in the worshipful company, which excited an extraordinary sensation of disapprobation, even in the ranks of those who supported the said company. He broached the opinion, that too good an education, and too much scientific knowledge, only rendered the surgeon-apothecary discontented with his lot—and, consequently, proved a disadvantage in his professional progress through life. This was so unsavoury a dose to the " march of intellect," that a spectator would have augured, from the countenances of the members, that each individual had that instant swallowed a large draught of salts, senna, and assafœtida! The poor examiner was obliged to eat up—his own physic—and we think a severer punishment could hardly have been inflicted.

Thus ended the first night's discussion, the remainder of the subject being adjourned till the succeeding week.

Although, in these debates, nothing was, or could be decided on, since the evil of apprenticeship was sustained by a parliamentary enactment, yet the debates of the evening tended to shew a powerful preponderance of sentiment and argument against the present system—and such developments of public feeling and opinion must pave the way to a repeal of the obnoxious laws, and a final correction of the evil. It is with the view of contributing our mite to this " consummation so devoutly to be wished," that we have presented a sketch of the Society's deliberations, and appended our own conclusions.

The adjourned meeting, on Saturday, the 13th October, was equally stormy, but not so interesting. The plan of study recommended by Dr. Hodgkin was, however, generally approved of, but, as it is already before the public through other channels, we shall not touch on it here.

Dr. Hodgkin should publish his paper, in order that it might circulate freely among the students.

---

### III.

### MEDICAL EDUCATION IN EDINBURGH.

By an able paper in a late number of our northern cotemporary, bearing the initials of the junior Duncan, we have now a view of the unfortunate litigation which subsists between the patrons and professors of the northern university. It appears, in short, that the intellectual citizens—that is, the worshipful companies of butchers, brewers, tanners, and weavers, have determined to shew the world that they can regulate the ACADEMIC CURRICULUM with as much exactness as they can cleave a chine of meat, stir up a vat of malt, curry the pelt of a calf, or ply the shuttle on a tartan petticoat! The town council consists of seventeen members of the merchants' company, and sixteen from " the incorporated trades." " By profession," says Dr. D. " none of these gentlemen are at all connected with letters, or necessarily in habits of communication with the learned—except the surgeon." Yet these are the men who are to legislate on all purely academic matters! It is pretty broadly hinted, that an individual of one of the incorporated trades—namely, the President of the College of Surgeons, is the prompter on this occasion. If he and his party carry the point against the SENATUS ACADEMICUS, they may give orders at once to one of the incorporated trades—the painter, to write, in large black letters, over the gate of the university—EDINA FUIT. The halls of that famous seat of medical lore will soon be as deserted as the ruins of Palmyra. This is the opinion of Dr. D. himself. "Were the honorable patrons to succeed in establishing their claims, the downfal of the university may be confidently predicted." What parent would send his child to be educated at a University whose fluctuating government (the members remain in office but one or two years) would be perpetually altering the regulations, so that no student could depend on the stability of the curriculum? Who would imagine that the patrons of the University tried all in their power to make the *new* regulations or statutes lately enacted *retrospective* on those who had previously been pursuing their studies! Every one knows that nothing is more unjust or impolitic than the retrospective operation of a new law. If we may judge by this sample of liberality, the *modern* Athens is in as tottering a condition as the *ancient!* Her walls, indeed, are not beleagured by the Turk and the Mameluke —but the foundations of her fame are sapped by the blind zeal, the turbulent passions, and the conflicting interests of her own citizens!

For the Senatus Academicus to maintain a tedious and expensive law-suit with the patrons, who had the public purse to draw upon, would have been madness; and, therefore, they wisely, as the lesser of two evils, appealed to the Crown, which has appointed a Commission to settle the disputed points. We sincerely hope, and confidently believe, that the Royal Commissioners will leave in the hands of the Senatus Academicus the management of all matters purely academical.

Dr. Duncan has made some excellent observations on the *preparatory knowledge* to be possessed by young men before they commence the study of medicine. By some medical reformers a most extravagant quantum of extra-professional learning and science is insisted on. They say it is absolutely necessary that physician, surgeon, and general practitioner, should be intimate with the Greek, Arabic, Latin—English, French, German, and Italian languages:—with moral and natural philosophy, logic, rhetoric, metaphysics, geometry, algebra, mechanics, hydrostatics, acoustics, optics, and, in short, with almost the whole range of human knowledge! Bless the literary and scientific noddles of these believers in the perfectibility of human nature! They seem to have no idea that life is bounded to 60 or 70 years, and that the study of only *one or two branches* of the healing art, such as pathology and therapeutics, would require a life of thrice the ordinary duration, to be even tolerably complete! Are we then to squander away a great proportion of our time in acquiring a knowledge of certain languages and sciences bearing so remotely on the practice of medicine, that it would be extremely difficult to *prove* them as auxiliaries at all; with the risk, nay, with the certainty of taking away time from the necessary study of the most vital and essential parts of our science? We must not legislate for those alone who are endowed with superlative talent. Every man is not a Crichton. We must adapt the scale of acquisition to the general run of mankind, leaving genius to outstrip its cotemporaries, and shine—

——— velut inter ignes,
Luna minores.

Upon these points, Dr. Duncan has made some excellent and pertinent remarks. He properly observes that the knowledge of languages, in itself, derives its chief utility from its facilitating the acquisition of useful knowledge. Let us take Greek, for example. This is the language of the fathers of physic, and that from which the terms of the medical art have been almost all borrowed. But, will any candid person aver that the useful portion of Hippocrates may not be as well learnt from the right as from the left hand columns of Vander Linden's edition—from the Latin as well as from the Greek? But then the terms are derived from the Greek. Be it so. If names are meant to signify things, we really see little advantage in knowing from whence that name is derived. Indeed, in innumerable instances, the derivation serves only to give us an erroneous notion of the nature of the thing so named. "It is," says Dugald Stewart, "in many cases, a fortunate circumstance when the words we employ have lost their pedigree;" for then, "the obscurity of their history prevents them from misleading the imagination, by recalling it to the objects or phenomena to which they owed their origin." Of what assistance will be the derivation of febris or typhus, in comprehending the nature of the malady? To be hot or to be soporose is not peculiar to fever or typhus. The derivation of inflammation would convey the idea of lighting a candle rather than the disease which it designates. What idea of erysipelas does the derivation of that word convey! In short, as Dr. Duncan has truly observed, "all attempts at descriptive terminology have utterly failed, and have impeded, instead of advancing the progress of knowledge." What has the terminology of Dr. J. M. Good done for the knowledge of diseases? Precisely what Dr. Duncan affirms—"it has had no other effect than to impede the dissemination of any addition to our knowledge that may be otherwise contained in his writings." God forbid that we should attempt to throw any slur on classical erudition. On the contrary, we believe that it eminently expands and liberalizes the mind; but to say that a knowledge of the Greek, or indeed of any other derivation of the names of diseases, conduces to a knowledge of the diseases themselves, is as false as it is absurd. If then the time or the capacity of the medical practitioner be inadequate to the acquirement of many languages, we would say, drop the Greek, rather than French, German, or Italian. Latin, for various reasons, is indispensable—but not so Greek. Natural philosophy, and the various branches of literature and science, are highly ornamental, but we question whether they conduce, in any very material degree, *and in a direct manner*, to the improvement of practical medicine. If it be true, and we believe it is, that the whole of the healing art consists in OBSERVATION, it follows that the study of classical learning, natural philosophy, polite literature, metaphysics, and the various mechanical arts, can only be considered as excellent and ornamental exercises for enlarging and strengthening the mind, thereby rendering it a more apt recipient for the purely medical knowledge that is to follow. But this, like every other good, is not without alloy. The delightful paths of literature and science form a great contrast with the dry, and often unsatisfactory, study of medicine. And we put it to those who are best able to judge, whether or not the mind inclines to those exercises and recreations which afford most pleasure and amusement, to the comparative neglect of the wearisome and difficult investigations of pathology and therapeutics. Had the late Dr. Good paid as much attention to the recent progress of pathology, as he did to poetry and metaphysics, he would not have been rejected by the College of Physicians. The writer of this article was examined at the College on the same day that Dr. Good was; and, in conversation, while pacing the long and sombre hall of old Warwick, he was astonished to find that the translator of Lucretius knew scarcely any thing of what had been done in the investigation of the seats and effects of diseases since the days of Morgagni! Here was an instance where the "pursuits of literature" and science had drawn the mind from, at least, *one* important branch of medical study. Were we disposed, we could adduce *living* instances of the same kind; but it is unnecessary. Nor are we unaware that many of the greatest ornaments of our profession were men of literature and science; but the example of John Hunter proves that science and literature are not *essential* to the most splendid attainments and discoveries in physiology, medicine, and surgery. As Dr. D. has well observed, "natural philosophy is the science of dead matter; the physician deals with living bodies, whose distinctive vital properties far overbalance the dead machinery." How far do the laws of mechanics assist the surgeon in reducing a dislocation—the laws of optics, the oculist—or those of acoustics, the aurist? Very little indeed! And these are the three branches of science which come more immediately into play in elucidating *physiology*.

As for the science of mind, we do firmly believe that the study of physiology and pathology (which are our own direct employments) throws more light on the nature of mind than all the metaphysical disquisitions that have ever been engendered in the brains of philosophers.

Upon the whole, we imagine it will be a very difficult and delicate matter to regulate the literary and scientific attainments of those who are destined for the general practice of their profession. That a competent education is desirable, and indeed necessary, no one will deny; but the *extent* of this preliminary education will be hard to fix. No doubt it is advantageous for the Fellows of the London College of Physicians to have a university education; but to expect this in all ranks and classes of medical society, is perfectly Utopian. What remuneration would nine-tenths of general practitioners have for such an expensive education? And would the study of classical literature, philosophy, and science, *in academic bowers*, qualify or adapt them for the drudgery of their profession among the different classes of society afterwards?

### IV.

### THE REGULATIONS AT APOTHECARIES' HALL.

That every candidate for a certificate to practice as an Apothecary, shall be required to possess a competent knowledge of the Latin language, and to produce testimonials of having served an apprenticeship of not less than five years to an Apothecary; of having attained the full age of twenty-one years, and being of a good moral conduct.

N.B. Articles of apprenticeship, where such are in existence, will be required; but, in case such articles shall have been lost, it is expected that the candidate shall bring forward very strong testimony to prove that he has served such an apprenticeship as the Act of Parliament directs.

He is also required to produce certificates of having attended not less than—

One course of lectures on materia medica and medical botany;

One course of lectures on chemistry;

Two courses of lectures on anatomy and physiology;

Two courses of lectures on the theory and practice of medicine: these last to be attended subsequently to the lectures on materia medica, chemistry, and to one course, at least, of anatomy.

N.B. No testimonial of attendance on lectures on the principles and practice of medicine, *delivered in London*, or within seven miles thereof, will render a candidate eligible for examination, unless such lectures were given, and the testimonial is signed by, a fellow, candidate, or licentiate of the Royal College of Physicians.

A certificate of attendance for six months, at least, on the medical practice of some public hospital or infirmary, or for nine months at a dispensary; such attendance to commence subsequently to the *termination* of the *first course* of lectures on the principles and practice of medicine.

N. B. Physicians' pupils, who intend to present themselves for examination, must appear personally at the beadle's office, in this Hall, and bring with them the tickets, authorising their attendance on such practice, as the commencement thereof will be dated from the time of such personal appearance.

The regulations relating to the order of succession in which the lectures on the practice of medicine, and the medical practice of an hospital or dispensary, are to be attended, are designed to apply to those students only who shall commence their attendance on lectures on or after the 1st of February, 1828; and all such persons are particularly requested to take notice, that unless they shall have strictly complied with such order of succession, they will not be admitted to an examination.

In addition to the course of study above required, and which is indispensably necessary, the candidates are earnestly recommended to attend one or more courses of lectures on midwifery, and the diseases of women and children, on the latter of which subjects, as an important part of medical practice, they will be examined.

The Court have determined, that the examination of the candidate shall be as follows:

1. In translating grammatically parts of the Pharmacopœia Londinensis, and Physicians' Prescriptions.

Should any doubt arise as to the Candidate's possessing a competent knowledge of Latin, he will be required to translate a passage, or passages, from some one of the easier Latin authors.

N. B. The Court are anxious to impress upon candidates a conviction of the necessity of a knowledge of the Latin language, because they have had the painful duty imposed on them of rejecting several persons, entirely from their deficiency in this important pre-requisite of a medical education.

2. In chemistry.
3. In the materia medica and medical botany.
4. In anatomy and physiology.
5. In the practice of medicine.

NOTICE.—Every person intending to qualify himself under the regulations of this Act to practise as an Apothecary, must give notice in writing, addressed to the clerk of the Society, on or before the Monday previously to the day of examination; and must, also, at the same time, deposit all the required testimonials at the office of the beadle, at Apothecaries' Hall, where attendance is given every day (except Sunday) from nine until two o'clock.

The Court will meet in the Hall every Thursday, where candidates are requested to attend at half-past one o'clock.

By order of the Court,
JOHN WATSON, Secretary.
London, Sept. 14, 1827.

Information relative to the business of this Court may be obtained of Mr. Watson, at his residence, 43, Berner's-street, between the hours of nine and ten o'clock every morning (Sunday excepted.)

*** It is expressly ordered by the Court of Examiners that no gratuity be received by any officer from any person applying for information relative to the business of this Court.

It will be seen that the principal changes in the above Regulations relate to the order of succession in which the various branches of medicine shall be studied. The great object is evidently to compel the student to pass two seasons —or, in other words, to study *two years* in London—a regulation which we cannot but approve, as we consider the ratio of medical education for the general practitioner as, even now, below what it should be. Those who urge that *an examination alone* should be the test for candidates of all kind in medicine, know nothing of the matter. There must be authentic proofs afforded of a *certain time* spent in the acquisition of knowledge, as well as of the identical spots where the knowledge was obtained, otherwise book-learning would, in a great measure, supersede the study of nature in the dissecting room, and at the bed-side of sickness. We do not say, indeed, that a more efficient examination is not necessary. Far from it. The mode of examination at the Royal College of Physicians is the worst of all calculated to test the candidate's qualifications—that in the College of Surgeons is better, but far from sufficiently strict—and we believe the same may be said of the Apothecaries' Company. But it is evident that the two last corporate bodies cannot rise *per saltum* to an *efficient* examination, till they can gradually introduce laws for an *efficient* preparatory study. This, they appear to be doing; and every step, however slow, by which they proceed, is entitled to the approbation of all who wish well to the profession.

We firmly believe that the increased period of study among students—the necessary enlargement of their education—the consequent acquisition of liberal ideas —and the aid of a spirited and upright press, will yet check the horrible system of demoralization and calibanism which has been lately instilled into their minds through the medium of a wicked press. The tide of honour and liberality has again turned, after the lowest ebb ever witnessed in modern times; and the results will soon be conspicuous. Our efforts shall not be wanting in the good cause.

### V.

### LUNACY—INSANITY—UNSOUND MIND.

The medical profession would really seem to be in a most critical situation. Scarcely is there a newspaper published, which does not attach some foul stain on the moral character or professional acquirements of medical men! Every one knows what the TIMES has lately said of the ignorance, and the mercenary character, of medical practitioners, as furnished by the representations of the LANCET and its partisans; and the MORNING CHRONICLE of Saturday, the 3d of November, plainly states that medical men, when called into a court of justice, are precisely like lawyers—namely that they will furnish arguments on any side of a professional question for which they are best paid! This caustic remark was called forth by the working of a commission of lunacy, issued against the Rev. Mr. Holmes, aged 77, and before which commission, a number of medical gentlemen were examined, and contradicted each other, as usual, in the most distressing manner.

It may not be useless to look a little into this evidence. Dr. Pennington, who had been physician to the Nottingham Lunatic Asylum for 15 years, had examined Mr. Holmes, and the following were the *facts* on which he grounded his opinion. "Breakfasted with Mr. H. Mrs. H. and Mr. Amor, her brother. A newspaper lay about, and the Dr. said, Do you read the newspaper, Sir? to which he replied, Oh yes, every day. Dr. P. remarked, They have been more than ordinarily interesting of late, on account of the melancholy death of Mr. Canning—to which he replied, O! Mr. Canning, poor man, is he dead? O! I did not know that he was dead; O! poor man. Knowing that he was acquainted with Mr. Benjamin Maddock, the surgeon, I said, Have you seen or heard any thing lately of your friend, Benjamin Maddock? Benjamin Maddock, said he, how does his brother John do? I said his brother John had been dead twenty years, to which he answered, O! poor man.—Witness afterwards saw Mr. H. alone. After some other conversation, I said, Sir, have you any child? He said, O no, no family, no children at all. I said, I understand, Sir, you've a daughter? He replied, O yes, I believe I have a daughter. Is she married? Yes, she is married, but has no family. Pray what is her name, the name of her husband? O, I don't know the name of her husband, and this answer he repeated several times. After a pause, he said, I think his name is Stevenson. I said, pray, Sir, how long has your brother been dead? To which he replied, I have a brother living, a druggist, at Leicester; he is not dead. I said, I understand he is dead, and you had property of your brother's. He said, O, I had a brother, who died some years ago, but he left me nothing. I said, Had you no property from Mrs. Chamberlain? Mrs Chamberlain, he said, is still living. I said, You have a large property, where is it situated? He said, I have property in Lincolnshire, but I do not know what is the name of the place. I said, I understood your income is considerable, what is it yearly? He said, I do not know how much my income is. Then you have a steward, I rejoined. He replied, no, indeed, not I, I have no steward; I do my business myself, and I go to Sleaford to receive my rents. Have you been there lately, Sir? to which he answered, I cannot recollect when I was there last. Another question was, Was Silcock a charwoman or a domestic servant? to which he replied, I do not know whether she is hired for a charwoman.—Do you know Mary Buckley? and to this he answered, I never knew Mary Buckley, no person of that name ever lived with me.—Another question was, Have you seen Dr. Arnold lately, or more than once? to this he said, he called on me once, but not the second time at all.—Have you seen Dr. Peach lately? I have not seen Dr. Peach lately, no not for a year.—Pray, Sir, what is your age? I am 77.—Such were the questions that Dr. Pennington put to him, and his replies. His manner was confused at times, and at times was trifling, and he generally ended his observations with a silly kind of laugh.—Witness considered his condition to be what is denominated *dementia*, or fatuity of mind, denoted in Mr. Holmes by the decay or abolition of the thinking faculty, which rendered him unfit for the ordinary purposes of life."

Dr. W. Arnold corroborated the above testimony, by stating it as his opinion, that "*mental imbecility, feebleness of mind, and a great want of memory*, were the chief features in Mr. Holmes's case." "For the management of pecuniary matters, he was totally unfit."

We shall pass over a number of non-medical witnesses, whose evidences were unimportant, though they tended substantially to confirm that of the two physicians above-mentioned. We now come to the great man of mind, on the other side of the question—namely, Dr. Haslam. He stated that, for 57 years past, his practice has been confined to diseases of the mind—that he had had sufficient means of knowing Mr. Holmes's state of mind—and that "he believed him to be a responsible being, completely so"—that he believed him not to be a lunatic—that "he did not think he was of *unsound mind* at all." Dr. Haslam was quite shocked at the idea of applying the term *unsound* to mind, as "*it has a tendency to spread the doctrine of materialism!*" Mr. Holmes's mind was perfectly sound—he was only "*non-compos*—that was the proper term." "If Mr. Holmes (a man acknowledged to be "*non-compos*") were to murder a man, *I would find him guilty*, and would *see him hanged after ! ! !*" "The mind cannot be said to be unsound, for, if we suppose the human mind an emanation from the Supreme Being, it is not susceptible of the ordinary corruptions of matter." "We can say an unsound tooth, an unsound horse, or unsound cheese; but we cannot say an insane tooth, an insane horse, or insane cheese, and, *therefore*, they are not convertible terms." On cross-examination, Dr. Haslam observed that "the expression *defective* mind relates to quantity:—The mind is incommensurable, but the *unsoundness of cheese* commensurable by the *number of mites*, and the extension of rottenness ! !" Notwithstanding the brilliancy of these metaphysical distinctions, the jury brought in a verdict of "unsound mind, and incompetency as to the management of his own affairs"—"partly from paralysis, and partly from old age"—but that he was not a lunatic.

The quibbling metaphysical jargon displayed in Dr. Haslam's evidence is perfectly sickening—and, after such a display, we do not much wonder at the caustic remarks of the Chronicle. But let us examine a little into the consistency of this great metaphysician, who is so shocked at the idea of the human mind, an *emanation from the Deity*, being "susceptible of the ordinary corruptions of matter." This emanation from the Deity is thus defined by Dr. Haslam himself, in his recent lectures.

"The *human mind* is not the progressive unfolding of *intellectual* germs, which nature first protrudes and subsequently expands; but a *structure* that is *reared, in its primordium*, by *casual excitations*, and, in its most important attainments, by the active exertions of the *individual himself*."—Lancet.

Now, if there ever was a doctrine or definition more completely, palpably, and unequivocally, that of *materialism* than the above, we acknowledge ourselves to be incapable of perceiving the difference between A and B. Such, however, is the doctrine delivered in *Bolt-court*, by a man, who, in a *court of justice*, cants forth the exalted doctrine, that the mind is an emanation from the Deity, and insusceptible of the ordinary corruptions of matter—that mind which he declares to be the nurseling of *casual excitations*, and dependent on the individual himself for all its more important attributes and attainments!

But again. Dr. Haslam, after pronouncing the Rev. Mr. Holmes to be "non compos," declares that, if the said *non compos* individual took away the life of another, he would not only find him guilty—but "*see him hanged afterwards ! !*" Gracious heaven! is it possible that such a *savage* declaration could issue from the mouth of a *medical philosopher* in the nineteenth century? Can we blame the Chronicle for affixing a sweeping stigma on the medical profession generally, when such evidences as the above come forth in courts of public justice! These are the ways in which a whole faculty is disgraced in the eyes of the public, by the clashing testimonies of men, from whom scientific truths are, or rather *were* expected, but whose evidences are *now* held in scorn and derision by judge and jury!

In fine, we have no hesitation in pronouncing the *opinions* of Dr. Haslam, on the above trial, to be *erroneous, quibbling, and ridiculous;* and that the verdict of the jury, which was in direct opposition to these opinions, was just, proper, and accordant with pathological science.

To maintain that the mind of man is *sound*, when all its more important faculties and manifestations are in utter decay, (as was the case with Mr. Holmes) is about as much as to say that a man is in perfect health and strength, when, as in the seventh Act of Shakspeare, he is —"*sans eyes, sans teeth, sans taste, sans every thing*." The organ of Mr. Holmes's mind was as incapable of performing its office, as is a harp of bringing forth harmonious sounds, when nine-tenths of its strings are broken.

We are sorry to observe that, in some cross-examinations of the other medical witnesses by the lawyers, certain confessions came out, not very complimentary to the "march of intellect" in the nineteenth century. Thus, when physicians were asked if they knew what were the sentiments of some of the most eminent medical writers, ancient and modern, on the subject of insanity, the reply was, that they had not perused any such writings, but that they had read "Haslam on Sound Mind!!" What must be the effect of such confessions on the minds of judge, jury, and auditors? We blush for the answer—but out it must come. The minds of medical men, of late, have been too much captivated with the seductive pleasure of seeing their brethren caricatured, scandalized, and defamed. The painful and laborious investigations of medical literature and science are quite disrelished after such piquant sauces. But the day of retribution is at hand—nay, it has already come! The professors of a learned and humane science are on the very brink of losing their character, and of being precipitated into the abyss of ignorance—if not the general detestation of their more liberal and enlightened neighbours! These are the effects of demoralizing publications and scandalous chronicles! They are coming home, with terrible retribution on the heads of the innocent as well as the guilty! The reaction is inevitable, and the ultimate consequences will be most salutary. Medical men will soon awake to their *own interests*, as well as to the respectability of their profession and the good of society at large. Another and a better æra is on the eve of bursting forth.

P. S. What between the technicalities of the law and the quibbles of physic, the relations of Mr. Holmes are likely to have merely the shells, while the lawyers and doctors devour the oysters. It appears that, in an appeal to the Chancellor, a flaw was discovered in the verdict of the jury, and a new commission is to be issued! Another council of Trent is to determine whether a man who is pronounced by both parties—even by Dr. Haslam, as *non compos*, be also of "unsound mind." Some of the Chancellor's observations are to us Chaldaic. This, however, we can understand. "If the jury had said directly that Mr. Holmes was of unsound mind, their finding would be direct and *proper*." But, in consequence, we suppose, of the fine spun theories which were sported by Dr. Haslam, the unlettered jury brought in Mr. Holmes as "afflicted with paralysis and old age, and consequently of unsound mind." This introduction of the *premises* was fatal to the *conclusion*, and the whole business is to be gone over again, in order to leave out the paralysis and old age! In law there is nothing certain but *ruin*—in physic, but *death*.

---

### VI.

### LUNATIC ASYLA.

In this inquiring age, it was not to be expected that the disgraceful manner in which pauper lunatics were *let out* to the *lowest* bidder, could long remain unstigmatized. Various investigations had taken place before committees of the House of Commons; and although facts of the most revolting nature came out, nothing was done in the way of remedy. The magistrates of the county of Middlesex at length took up the business, and, after a somewhat stormy discussion, it was carried, almost unanimously, that a county lunatic asylum should be erected for insane paupers, in order to supersede the disgraceful system of sending them out, at 14 or 15 shillings a week, to starve, rot, and become incurable, in certain of those wretched asyla, ycleped "private madhouses." In this discussion, we are sorry to observe that transactions came to light not very well calculated to enhance the character of the medical profession, whether we look to the highest body or to the humbler members of that profession. Thus, it was proved by Serjeant Pell, that, although the houses for the reception of lunatics are (or rather are said to be) under the inspection and control of the Royal College of Physicians, yet it appeared from evidence before the House of Commons, "that that learned Body either did not possess, or did not exercise any efficient check and control for the correction of the abuses which prevailed in these establishments." The learned commissioners visited these asyla once or twice a year—they granted licences to whoever applied for them, "no matter how unfit the persons making the application might be—no matter how gross their former misconduct." Yes, yes! the *licensing* trade has been an excellent one at the College—the fitness or unfitness of the persons licensed was quite another thing! The price of the licence was the 'main chance;" but, for the character of madhouse-men or licentiates, the College was not responsible! Well might Serjeant Pell observe that, if the College had no power or inclination to *withhold* licences, "wherefore was the authority of *granting* licences at all given to them." Wherefore, indeed! But can any rational being doubt, for a moment, that, were the College commissioners to do their duty in *detecting and reporting abuses*, they would be supported by the proper authorities, and licences would not only be withheld but *withdrawn*, where delinquencies obtained? The truth is, that, because the Act of Parliament *is* defective, the commissioners, like commissioners in general, considered the routine of their inspection as a matter of course, and a mere form of doing a certain quantum of duty, annually, without any corresponding return of utility to the public or to humanity!

But we are compelled to advert to a disclosure which will long rank high among the indelible stigmata which the present demoralizing influence of the medical press has given rise to, and affixed on the medical profession.

Mr. Jessop, in adverting to the parliamentary reports, stated, that the evidence given before the committee presented such a lamentable exhibition "of evasion, subterfuge, and downright falsehood," as he had never before witnessed in the course of his professional life. A surgeon who attended a celebrated establishment for insane paupers, as assistant to another surgeon, asserted, point blank, that his superior or principal kept a regular book of the cases, on which book he pronounced a high eulogium. Yet, when the principal, Mr. D——, was examined, he declared, that the whole statement of his friend was a romance, without a particle of foundation, as he never kept any book of the kind!! The auxiliary surgeon described, with great minuteness, the shape, size, and arrangement of this register, the existence of which had no other "local habitation" than his own brain!

And can we wonder at such exhibitions, when the medical press, for several years past, has taught, by precept and example, the various methods of cultivating the organ of *mendacity*, with the greatest degree of perfection and success? Can we wonder that men of weak and bad principles should readily give way to such propensities, when they see them practised with great profit and advantage, by directors of the medical press? Will it be believed, that there are men in this great metropolis, who rank high in medical science, but who are wicked enough to expend considerable sums in the reward of those who can invent the greatest, but, at the same time, the most plausible falsehoods, against the brightest characters in the medical profession? For the honour of human nature, we hope this will not be believed—yet such, we are credibly informed, is a certain fact! The days of Sparta are returning, when thievery will be stigmatized *only* when it is awkwardly performed!

### VII.

### WONDERFUL VIRTUE OF VENESECTION.

The Medico-Chirurgical Society has revived from its bed of sickness or of death, and flourished. The Lancet begins once more to report, and modestly attributes the resuscitation of the Society to the vivifying influence of ITS REPORTS!! The modesty and the truth of this passage are quite on a par. There is just as much veracity in this statement as in the statements in general contained in the *leading articles* of that instrument, the beauties of which we shall now have pretty frequent opportunities of portraying. It has now come out,* that the EDITOR is merely the PROPRIETOR, and that all the flaming articles about reform—in short, the whole of the *leading articles*, were written by hireling scribes, who cared no more for the profession than for the inhabitants of the moon!! By the way, we have a word or two to say as to the right or the propriety of these reports of societies. We conceive, then, that the papers read at a society which publishes the said papers, in the form of transactions, are, or ought to be, sacred; and no journalist has any right to publish reports of them before the society. The case, we conceive, is very different with those societies which do not publish their transactions—and with those debates which take place in consequence of the reading of papers afterwards to be published. These debates are no man's property. They are words spoken—and, if fairly condensed, the publication of them is praiseworthy and free from objection. Till very recently, debates were discouraged in the Medico-Chirurgical Society,

* Vide DISSECTOR, No. 7.

and nearly two years ago we publicly urged the Society to adopt measures for promoting *viva voce* discussions. These measures have been adopted—and hence one cause, at least, of the resuscitation of the Society. One other cause, we allow, was the Billingsgate abuse lavished on the society, and the prediction of its dissolution, by the junto of the LANCET. This abuse and this prediction had precisely the same effects as the strenuous workings of the Lancet had on the Bartholomew students. They produced their antidote.

---

A LANCET and a FALSEHOOD have long been so completely synonymous that it is hardly worth while to rebut the mendacious assertion that we have discouraged the publication of hospital reports. Ever since the commencement of our Journal, we have never ceased to urge the example of the continental hospitals, where intelligent elèves are appointed to record and publish cases. But we always did, and always will condemn the publication of garbled, distorted, and falsified reports, as manufactured for the LANCET —a journal which pays the highest price to that reporter who shews most alacrity and ingenuity in falsifying all cases that happen under any physician or surgeon not of the junto! *This kind of publication* we shall not fail to hold up to the scorn and detestation of mankind—while every fair and correct hospital report shall receive our meed of approbation.

---

### VIII.

### COLLEGE OF PHYSICIANS *versus* DR. HARRISON.

Many versions of this *versus* have been given to the public, without any authority —and, what is worse, without any foundation in truth. It has been asserted that the prosecution has been given up by the College. It has never yet commenced. The latest information which we have been able to obtain (up to the 8th Dec. when this sheet went to press) is, that a committee had been appointed by the College to collect evidence of practice against Dr. Harrison; but that it was very uncertain when the proper legal proof would be obtained. Many weeks ago, the lawyers of the adverse parties had a meeting, when the College solicitor asked for *voluntary* proof of practice on the part of Dr. Harrison. This was refused. For our own parts, had we been in Dr. Harrison's place, we should, after what had passed, have given up the admission of practice, in order to bring the question to a legal issue. The only reasonable excuse for withholding this admission, is, to try whether the College can bring home the *necessary legal proof of practice,* against any man. As the prosecution must hinge entirely on the acts growing out of the charter of Henry VIII. and as it is there decided that a man must practise (de die in diem, of course) for the space of a month, before he is finable, so we apprehend that it would be extremely difficult to bring home such proof against Sir Henry Halford himself. If we were on the jury, (for a jury and not a judge must decide as to the proof of Dr. H's practice,) we should certainly interpret the statute as "*uninterrupted* practice for 28 days"— and it is just possible, whatever the lawyers may opine, that a jury will look at the act or statute with the eye of common sense, and not under the influence of corporate pride or legal quibbling. On this principle, Dr Harrison is probably right in trying the issue of the preliminary step; for if the College demur in prosecuting, on the ground that evidence cannot be obtained against a man in the receipt of two or three thousand a year, as a physician, then, of course, no man can be prosecuted, and the matter, so far as Dr. Harrison is concerned, will drop. But the physicians of England and Scotland should not let the question fall to the ground. They should, one and all, unite in a petition, first to the College, and if rejected there, to the Legislature, praying for an inquiry into the state of this part of the profession. They should form no cabals—give themselves no designations—deviate not an inch from public, manly, and constitutional proceedings, for the redress of their grievances. Our own advice would be, for physicians, surgeons, all classes of medical society, to unite in one general petition for parliamentary inquiry into the state of the profession, so that the present disgraceful chaos—this

"Monstrum horrendum informe,"

may be re-organized and set on a level with the faculty of physic and surgery in other nations. We shall quickly return to the subject again.

